# Differentiation of arterial and venous neurovascular conflicts estimates the clinical outcome after microvascular decompression in trigeminal neuralgia

**DOI:** 10.1186/s12883-020-01860-8

**Published:** 2020-07-14

**Authors:** Sebastian Müller, Eya Khadhraoui, Ali Khanafer, Marios Psychogios, Veit Rohde, Levent Tanrikulu

**Affiliations:** 1 Departments of Neurosurgery, Georg-August-University Goettingen, Robert-Koch-Str. 40, 37075 Goettingen, Germany; 2 Departments of Neuroradiology, Georg-August-University Goettingen, Göttingen, Germany

**Keywords:** Trigeminal neuralgia, B-SFFP, FSE, TOF, MVD

## Abstract

**Background:**

Balanced Steady State Free Precession (b-SSFP) sequences and the newly developed Fast-Spin-Echo (FSE)-sequences enable an optimized visualization of neurovascular compression (NVC) in patients with trigeminal neuralgia (TN). Arterial conflicts are mostly associated with a favorable outcome of microvascular decompression (MVD) compared to venous conflicts. An additional Time-of-Flight (TOF) angiography provides the differentiation between offending arteries and veins and a precise counselling of the patient concerning postoperative pain relief. The goal of this study was to analyze the reliability and impact of the combination of highly-resoluted MRI techniques on the correct prediction of the vessel type and the estimation of postoperative outcome of microvascular decompression (MVD).

**Methods:**

In total, 48 patients (m/f: 32/16) underwent MVD for TN. All the preoperative imaging data (T2: b-SFFP and FSE, MRA: TOF) were compared to the intraoperative microsurgical findings during MVD. b-SFFP was available in 14 patients, FSE in 34 patients and an additional TOF sequence was available in 38 patients (9 times in combination with b-SSFP, 29 times in combination with FSE). The patients were categorized into four subgroups: 1) NVC negative, 2) venous NVC, 3) arterial NVC, 4) combined arterial and venous NVC. The preoperative MRI findings were compared to the intraoperative morphological findings. Postoperative pain relief was quantified by the Barrow Neurological Institute pain score.

**Results:**

Twenty-five purely arterial NVC, 9 purely venous NVC and 5 combined arterial and venous NVC were detected by MRI. In 9 cases NVC was absent on MRI. Overall, the MRI findings correctly predicted the intraoperative findings in 91.7% of the 48 patients. The percentage of correct prediction increased from 80 to 94.7%, when TOF angiography was adjoined.

**Conclusion:**

The visualization of the trigeminal nerve using sequences such as b-SSFP or FSE in combination with TOF angiography enables an optimized delineation of arterial and venous neurovascular conflicts and may allow a more reliable differentiation between veins and arteries, resulting in superior prediction of postoperative pain relief compared to T2 imaging data alone.

## Background

Classical trigeminal neuralgia (TN) is a facial pain disorder caused by distinct vascular compression of the trigeminal nerve mostly at the root entry zone [1–4]. The vascular compression caused by arterial or venous loops might induce focal demyelination at the transition between central and peripheral myelin sheath, which can lead to ectopic impulses and ep-haptic transmission, so that facial pain attacks can be triggered [5]. Diagnosis is obtained by clinical history and symptoms. Microvascular decompression (MVD) by Jannetta is the preferred surgical treatment option in patients with medication-resistant facial pain [5–7]. The identification of the vessel type for the estimation of postoperative outcome of microvascular decompression is very crucial, while the presence of major morphological neurovascular changes on the trigeminal root at the site of the conflict plays an important role to predict the positive outcome. The site of conflict, which usually is localized at the trigeminal nerve root can be variable and may extend to the distal cisternal segments of the trigeminal nerve. In order to provide sufficient postoperative pain relief, MVD should not only include the decompression of the trigeminal root entry zone by an offending vessel, because vascular contacts at the peripheral nerve segments may also cause neuralgic symptoms. Preoperative imaging with highly-resoluted magnetic resonance imaging (MRI) is recommended for the confirmation of neurovascular compression (NVC) [8–11]. Since the outcome of arterial and venous conflicts differ in larger studies [6, 12], a reliable prediction of the vessel type is desired. On the preoperative highly T2-weighted sequences, the superior cerebellar artery was seen as the most common offending vessel in more than 60% of cases [13]. Veins are usually coursing laterally to the trigeminal REZ and can be outlined in the TOF sequences by lower intravascular signal intensity. The main goal of this article was to analyze the impact of the combination of highly-resoluted MRI techniques (T2: b-SSFP/FSE and TOF-angiography) on the prediction of arterial or venous pattern of the offending vasculature with the focus of on the estimation of postoperative pain relief in trigeminal neuralgia.

## Methods

### Clinical data

In total, 48 patients (m/f: 32/16) underwent MVD for TN. All patients were admitted between the years 2012 and 2018 into the Department of Neurosurgery of the University of Goettingen. All recruited patients hat unilateral classical trigeminal neuralgia, while two patients had a coincidence with multiple sclerosis and another two had patients showed persistent unilateral trigeminal pain. The study design was retrospective. The clinical outcome was evaluated by the Barrow Neurological Institute Pain Score (Table 1) [14]. The preoperative score and the six-month follow-up-data were collected from all patients. It was converted in percentages for a clear representation. The mean age at the time of MVD was 59.63 ± 13.56 years. In 35 (72.9%) cases the right side was symptomatic. The clinical data are shown in Table 2.

**Table 1:** Barrow Neurological Institute Pain Intensity Score

Score	Description	Pain	Relief
I	No pain, no medication	0	100
II	Occasional pain, no medications required	25	75
III	Some pain, adequately controlled with medications	50	50
IV	Some pain, not adequately controlled with medications	75	25
V	Severe pain or no pain relief	100	0

**Table 2:** Demographics and clinical data

**Total number of patients**	**male**	**female**	**average age**	**Affected side:** **right**	**Affected side:** **left**
48	32 (67%)	16 (33%)	59.63 ± 13.56	35 (73%)	13 (27%)

### Imaging

**Fig. 1:**
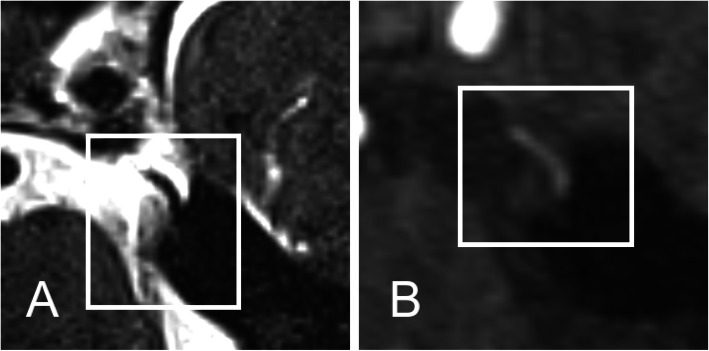
**a** NVC of SCA and CN V: (A) MRI-SPACE (B) MRI-TOF. A: MRI-SPACE sequence with neurovascular compression of the trigeminal root entry zone by a loop of the superior cerebellar artery. Note the highly-resoluted T2 enhanced sequence with hyperintense cerebrospinal fluid space containing hypointense vessels and cranial nerves. **b** MRI-TOF (time of flight angiography) showing the hyperintense arterial flow within the superior cerebellar artery.

The MRI data were categorized into four subgroups (Fig. 1):

T2 (b-SSFP and FSE) without TOF: 10 patients.

T2 (b-SSFP and FSE) with TOF: 38 patients.

Thirty-One 3-Tesla-MRI-examinations (PrismaFit®, Siemens Erlangen, Germany) and seventeen 1.5-Tesla-MRI-examinations (AvantoFit®, Siemens Erlangen, Germany) were available. The neuroimaging protocol consisted of the following details:

MR-T2: Acquisition time 8:26 ms, Voxel size 0.4mm^3^, Section thickness 0.4 mm, TR 7.48 ms, TE 3.23 ms, flip angle 45°. MR-TOF: Acquisition time 15:51 ms, Voxel size 0.4mm^3^, Section thickness 0.4 mm, TR 21 ms, flip angle 18°.

The analysis was blinded to the clinical and the microsurgical findings. The existence of a neurovascular compression at the root entry zone of the regarding trigeminal nerves were analyzed in the high-resolution MRI imaging. Arterial and venous vessels could be differentiated by the imaging data and the microneurosurgical experience. Veins usually couse laterally to the trigeminal nerve and arteries course on the mediolateral route.

### Neurosurgical technique

All patients underwent MVD. If a vascular conflict was found, the offending vascular loop was mobilized from the nerve and Teflon was interposed between the offending vessel and the trigeminal nerve [5, 7]. The patients with absent NVC in the preoperative imaging but severe clinical symptoms of typical neuralgia where thouroughly counselled for the surgical exploration.

The surgical reports were analyzed and the patients were assigned to the following groups (Table 3):

1. No vascular conflict found.
2. Purely venous compression found during MVD.
3. Purely arterial compression found during MVD.
4. Combined venous and arterial compression found during MVD.

**Table 3:** Contigency table with the corresponding categories

**Patient Group No**	**Neurovascular Compression**
***I***	*NVC negative*
***II***	*Pure venous NVC*
***III***	*Pure arterial NVC*
***IV***	*Combined arterial and venous NVC*

### Statistical analysis

For statistical evaluation the program Statistica (data analysis software system), version 13 (TIBCO Software Inc., Palo Alto, *California, USA*) was applied. *P*-values below 0.05 were defined as statistically significant. A two-sided t-test and a Mann-Whitney-U-Test of all aforementioned groups were used.

## Results

### Imaging

The imaging of the neurovascular conflicts were analysed by two independent neuroradiologists. Tables 4 and 5 show the distribution of the found neurovascular conflicts using the T2-weighted imaging alone and in combination with the time-of-flight MR angiography. Twenty-five purely arterial NVC, 9 purely venous NVC (superior petrosal vein) and 5 combined arterial/venous NVC were detected by MRI. In 9 cases NVC was absent on MRI. The causative arteries in the MRI data were diagnosed as: SCA *n* = 28 (93.33%), AICA n = 2 (6.67%), while both AICA loops were compressing the trigeminal nerve in combination with the SCA.

**Table 4:** The consistency of the T2-MRI without TOF and the microsurgical findings during MVD

	***NVC negative***	***Venous NVC***	***Arterial NVC***	***Arterial and venous NVC***
***MRI*** ***(T2 without TOF)***	*2*	*3*	*5*	*0*
***MVD***	*1*	*2*	*7*	*0*
***Consistency***	*%50.00*	*%66.00*	*%71.41*	*%100.00*

**Table 5:** The consistency of the combination of T2-MRI with TOF and the microsurgical findings during MVD

	***NVC negative***	***Venous NVC***	***Artery only***	***Both***
***MRI (T2&TOF)***	*7*	*6*	*20*	*5*
***MVD***	*7*	*6*	*22*	*3*
***Consistency***	*%100.00*	*%100.00*	*%90.91*	*%60.00*

### Microsurgical results

In 29 MVDs a single causative artery (SCA: n = 28, AICA: *n* = 1) and in 8 surgeries an affecting vein (the superior petrosal vein) were detected. Peri- and postoperative complications did not occur. In three patients a combination of arterial and venous NVC was found.

In one patient with absent NVC in imaging, intraoperative exploration harbored a bony spur of the petrous bone, which was affecting the trigeminal nerve, which was removed by drilling of the spur.

### Analysis of postoperative pain relief

**Fig. 2:**
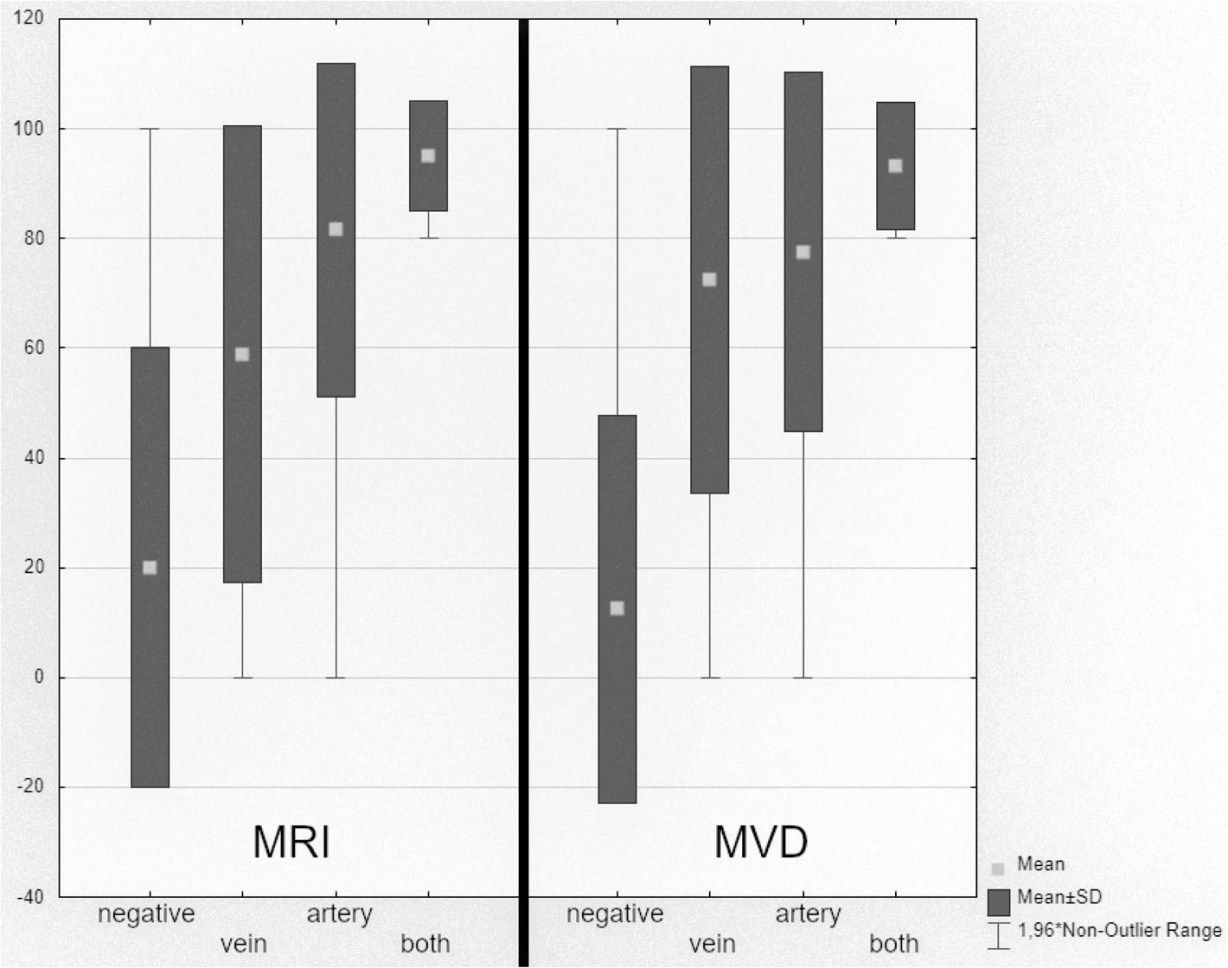
Outcome (pain relief) in percentage of patients grouped by MRI (left) and during microvascular decompression (MVD) (right). Outcome of patients grouped by MRI and surgical report. The figure demonstrates the outcome (pain relief in percentage) of the classified groups separated by MRI (left) and microsurgical observations during MVD (right) in the following order: 1 - no vascular conflict; 2- purely venous compression; 3 - purely arterial compression; 4 - combined venous and arterial compression

**Fig. 3:**
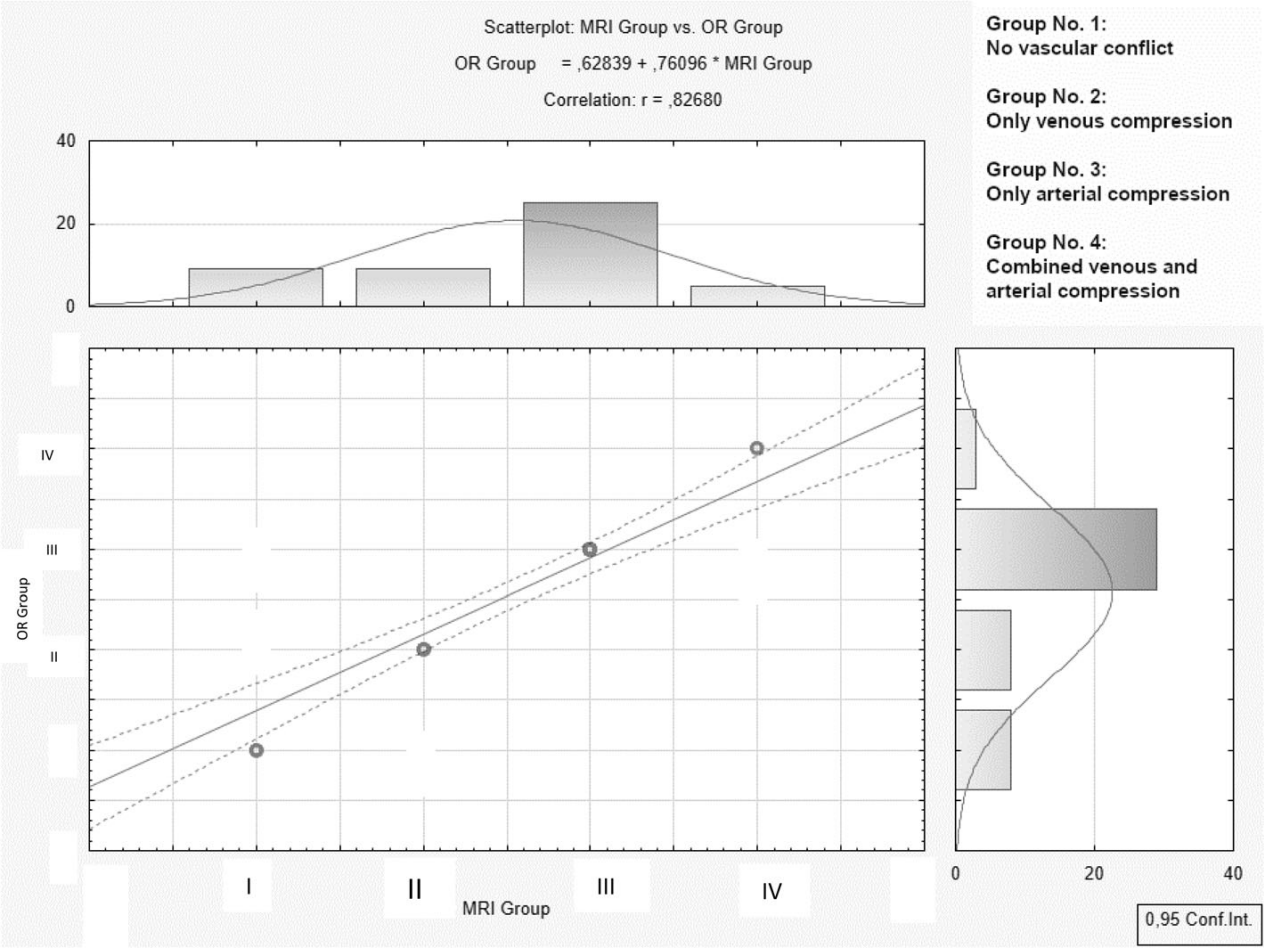
Correlation between the MRI findings and microsurgical observations during MVD. The graph shows the classification of the vascular conflict created by MRI on the x-axis as well as by microsurgical groups on the y-axis in the following order: 1 - no vascular conflict; 2- purely venous compression; 3 - purely arterial compression; 4 - combined venous and arterial compression. The MRI findings correctly predicted the intraoperative findings in 91.7% in the 48 patients. The percentage of correct prediction increased from 80 to 94.7%, if TOF angiography was combined to the T2 sequences. Pearson’s correlation between the MRI data and surgical findings was significant: r = 0.827, *p* < 0.05. With the combination of T2 sequences and TOF angiography the prediction rate of postoperative pain relief was significantly enhanced

Overall, the MRI findings correctly predicted the intraoperative findings in 91.7% in the 48 patients. The percentage of correct prediction increased from 80 to 94.7%, if TOF angiography was combined to the T2 sequences (Figs. 2 and 3, Table 6).

**Table 6:** Consistency between the findings of NVC in the MRI data (highly-resoluted T2 alone and T2 plus TOF angiography). The percentage of correct prediction increased from 80 to 94.7%, if TOF angiography was combined to the T2 sequences

	***MRI (T2 alone)***	***T2 + TOF***	***Sum***
***NVC confirmation in MRI***	*8*	*36*	*44*
***NVC confirmation during MVD***	*10*	*38*	*48*
***Consistency***	*80.00%*	*94.74%*	*91.67%*

38 of 48 patients benefited from the MVD with 83.94% reduction of their facial pain in a 6-months follow-up after surgery. In 10 cases no effect was shown. Only two of 8 patients with an absent NVC benefited from the explorative surgery. In 6 out of 10 patients NVC was absent. Two of the remaining four patients had a conincidence with multiple sclerosis and the other two had persisting unilateral trigeminal pain (Table 7).

**Table 7:** Description of the outcome in the four groups of patients

Patient groups	Number of patients	Number of pat.	Clinical details		Postoperative pain relief directly after MVD	Postoperative pain relief after 6 months follow-up
**NVC positive**	38 (79%)		Classical trigeminal neuralgia		100%	83,94%
**NVC negative**	10 (21%)	6	Classical trigeminal neuralgia		0%	0%
		4	2	MS		
			2	PUTP		

Abbreviations (*MS* multiple sclerosis, *PUTP* persistent unilateral trigeminal pain)

**Fig. 4:**
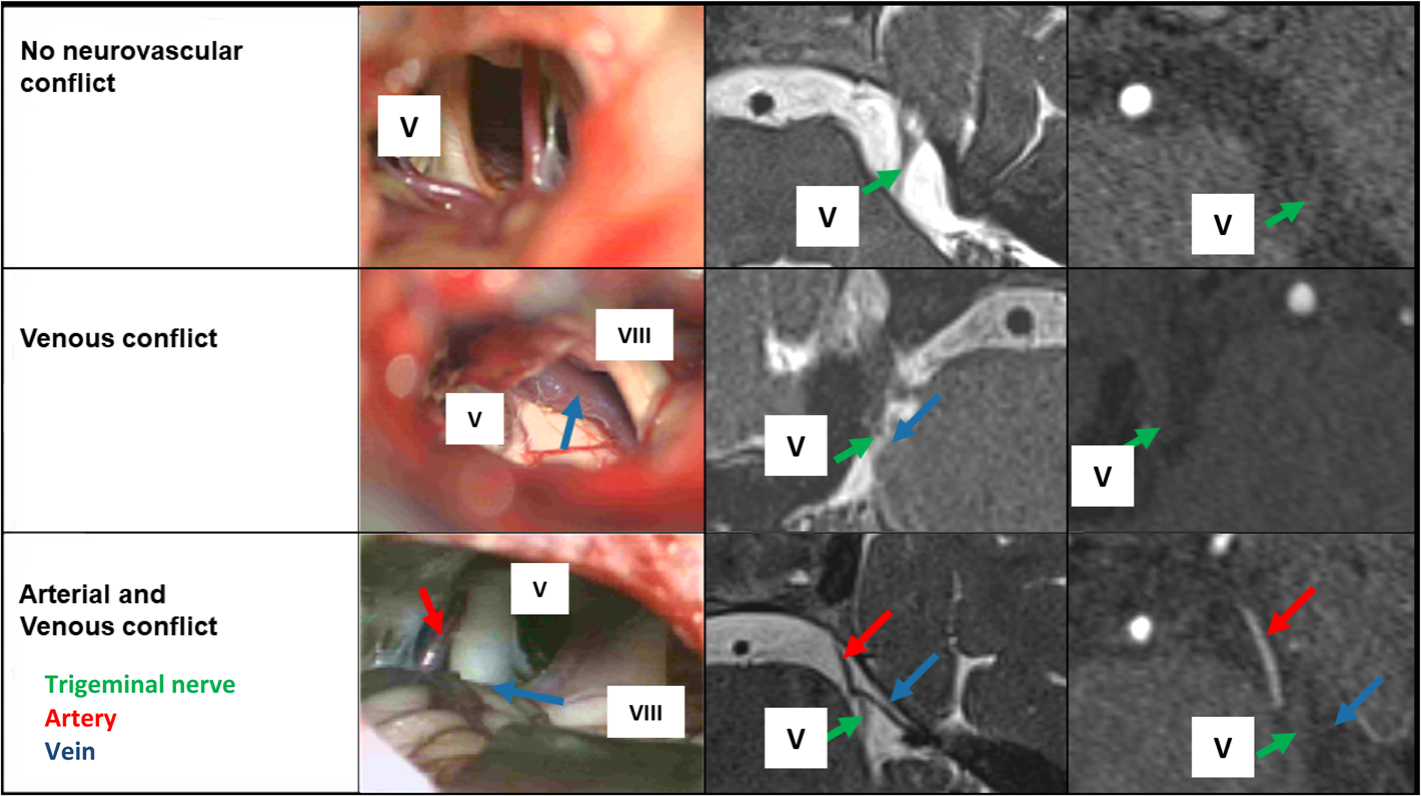
Comparison of microsurgical anatomy, MRI-SPACE and MRA-TOF (V: trigeminal nerve, VIII: vestibulocochlear nerve)

MRI allowed a good prediction of the intraoperatively confirmed vessels (See Table 4). The additional TOF-Sequence improved the differentiability of veins and arteries to an optimal level (Table 5). Pearson’s correlation between the MRI data and surgical findings was excellent: r = 0.827, *p* < 0.05 (Fig. 3). With the combination of T2 sequences and TOF angiography the prediction rate of postoperative pain relief was significantly enhanced (Figs. 2 and 4).

## Discussion

MVD shows the highest success rates in patients with classical trigeminal neuralgia [5]. 80% of the patients with proven NVC are pain-free after surgery [6, 7]. This article showed that with the combination of highly-resoluted T2-imaging and MR-angiography allows a better differentiation of arterial and venous neurovascular compressions compared to T2-imaging alone. Our study showed that with the combination of different imaging modalities such as T2-sequences and MR angiography by time-of-flight results in enhancing of the visualization of the neural and vascular delineation in the preoperative workup. Hence, a better prediction of postoperative pain relief is possible.

The majority of studies shows that microsurgically observed arterial compressions are associated with a superior clinical outcome compared to patients with venous conflicts [15, 16], which was also shown in this article. Other factors were widely discussed in the last years. The distance of nerve and the causative vessels, the abnormalities of the trigeminal nerve and the degree of compression were examined and classified [8, 10, 12, 17, 18]. Our approach was to clarify the high amount of possible MRI sequences by the application of the combination of high resolution MRI sequences. In our number of patients, the standardized use of b-SSFP/FSE - and TOF-Sequences was sufficient for the diagnosis and categorization. MRI sequences with gadolinium were not considered. b-SFFP was available in 14 patients, FSE in 34 patients and an additional TOF sequence was available in 38 patients (9 in combination with b-SFFP, 29 times in combination with FSE). This heterogenity of preoperative magnetic resonance imaging data surely influenced the impact on the validity of the neurovascular findings, while with the microneurosurgical experience a certain assignment of the neurovascular details was enabled in all cases. Comparing our study with the literature a standard preoperative imaging workup by high-resoluted T2 and MR-angiography should be implemented in all patients with trigeminal neuralgia. While the male preponderance in trigeminal neuralgia is unusual, in our study more male patients were admitted with trigeminal neuralgia than female patients, whereas refractory patients were not enrolled consecutively.

Standard MRI has not proved to be sufficient for reliable interpretation of the complex anatomy of the cerebellopontine angle [10]. High-resolution MRI sequences have been found to be better for depicting the fine trigeminal neurovascular anatomy [10]. High-spatial resolution three-dimensional T2-weighted MRI allows a fine anatomical analysis of the vasculo-nervous structures in the cerebellopontine angle cistern; its limitation is the absence of signal differentiation, not only between arteries and veins but also between vessels and nerves [10]. Time-of-flight MR angiography provides good visualization of the arteries in hyperintense, contrasting with the cerebrospinal fluid in hypointense [10]. Nerves are visible, but they are difficult to distinguish because of their intermediate signal [10]. Veins, because of their low flow, usually are not visible, especially if a band of presaturation filter is applied [10].

The limitations of our study is its retrospective design and limited number of treated patients. 9 patients without preoperatively shown neurovascular compression in the high-resolution imaging underwent MVD. These patients were informed that on imaging a clear neurovascular conflict was not detected and the patients were counselled that open surgery for exploration is a diagnostic and possible therapeutic choice, while the postoperative success rates surely are not as high as in patients with preoperatively confirmed NVC. Surely this subgroup of patients was counselled on lesional therapeutic options such as radiosurgery and rhizotomy; it was their clear request to undergo microsurgery. Further there are potential additional investigations available such as grading the conflict or use of diffusion-tensor imaging, which should be used in unclear preoperative imaging in order to hardening the indication for MVD. A grading system to categorize the morphological course of the offending vessel and its impact on compressing the root entry zone of the trigeminal nerve should be evaluated in further studies in order to estimate the degree of conflict [12]. We did not observe any kind of postoperative complications such as hearing loss, facial hypesthesia, facial weakness or infection.

A detailed multidisciplinary analysis of the patients should be performed by the neurologist, neuroradiologist and the neurosurgeon in order to optimize the surgical result for pain relief.

Neurovascular compression syndromes such as trigeminal neuralgia, hemifacial spasm and glossopharyngeal neuralgia are characterized by hyperactive cranial nerve dysfunction [19–22]. This results from a compression of the root entry or exit zone of cranial nerves by small vessels at the surface of the brainstem. Intraoperative observations showed distinct neurovascular compression at the level of the regarding REZ of the cranial nerves. Because the relations of structures in the posterior fossa are very complex, a comprehensive spatial understanding can be helpful for diagnosis and preoperative planning of surgery. However, an appropriate imaging was difficult in former times because of the small size of neurovascular structures. Image data providing a delineation of the target structures were obtained with magnetic resonance imaging. Several techniques have been examined and the strongly T2-weighted sequences such as CISS, SPACE, FIESTA, FSE turned out to be the most suitable for diagnosis, preoperative and perioperative applications. It reveals high contrast of nerves and vessels embedded in the CSF space, making even small structures visible with a diameter of less than 1 mm. Another improvement in the delineation of vessels is the use of time-of-flight sequence, MR-TOF [10].

With our article we strongly recommend to combine the information of different MR sequences, mainly combining T2-weighted data and MRA to obtain more comprehensive neurovascular representations of each individual patient anatomy. The vascular information taken from MRA for the purpose of an optimized differentiation of arterial loops combining with the T2 data led us to a more enhanced estimation of the anticipated microsurgical findings in order to enhance the success for postoperative pain relief optimizing the clinical outcome after microvascular decompression.

## Conclusion

Differentiation between arteries and veins could estimate the postoperative clinical outcome of the patients with trigeminal neuralgia, especially in a very short follow-up. The combination of high-resoluted b-SSFP- or FSE-Sequences and MR-angiography (TOF) sequences enables a clear differentiation between venous and arterial neurovascular compression in patients with trigeminal neuralgia. This essential preoperative information provides an optimized patient consultation and estimation of the outcome. A better prediction of the intraoperative microsurgical anatomy may also support the operative process of microvascular decompression by neuroimaging.

## Data Availability

Available with the corresponding author.

## Abbreviations

AICA: Anterior inferior cerebellar artery
b-SSFP: Balanced Steady State Free Precession
CISS: Constructive Interference in Steady State
FSE: Fast Spin Echo
MRI: Magnetic resonance imaging
MVD: Microvascular decompression
NVC: Neurovascular compression
REZ: Root Entry Zone
PICA: Posterior inferior cerebellar artery
SCA: Superior cerebellar artery
SPACE: Sampling Perfection with Application optimized Contrasts using different flip angle Evolution
TN: Trigeminal Neuralgia
TOF: Time of Flight
